# Factors controlling surface ozone in the Seoul Metropolitan Area during the KORUS-AQ campaign

**DOI:** 10.1525/elementa.444

**Published:** 2020

**Authors:** Heejeong Kim, Junsu Gil, Meehye Lee, Jinsang Jung, Andrew Whitehill, James Szykman, Gangwoong Lee, Deug-Soo Kim, Seogju Cho, Jun-Young Ahn, Jinkyu Hong, Moon-Soo Park

**Affiliations:** * Department of Earth and Environmental Sciences, Korea University, Seoul, KR; † Center for Gas Analysis, Korea Research Institute of Standards and Science, Daejeon, KR; ‡ US EPA, Research Triangle Park, Durham, NC, US; § Department of Environmental Sciences, Hankuk University of Foreign Studies, Yongin, KR; ‖ Department of Environmental Engineering, Kunsan National University, Kunsan, KR; ¶ Seoul Metropolitan Government Research Institute of Public Health and Environment, Gyeonggi-do, KR; ** Department of Climate and Air Quality, National Institute of Environmental Research, Incheon, KR; †† Department of Atmospheric Sciences, Yonsei University, Seoul, KR; ‡‡ Research Center for Atmospheric Environment, Hankuk University of Foreign Sturdies, Yongin, KR

**Keywords:** KORUS-AQ, Air quality, Ozone, NO_x_ and VOCs, PM_2.5_, SMA (Seoul Metropolitan Area)

## Abstract

To understand the characteristics of air quality in the Seoul Metropolitan Area, intensive measurements were conducted under the Korea-United States Air Quality (KORUS-AQ) campaign. Trace gases such as O_3_, NO_x_, NO_y_, SO_2_, CO, and volatile organic compounds (VOCs), photochemical byproducts such as H_2_O_2_ and HCHO, aerosol species, and meteorological variables including planetary boundary layer height were simultaneously measured at Olympic Park in Seoul. During the measurement period, high O_3_ episodes that exceeded the 90 ppbv hourly maximum occurred on 14 days under four distinct synoptic meteorological conditions. Furthermore, local circulation such as land–sea breeze and diurnal evolution of the boundary layer were crucial in determining the concentrations of precursor gases, including NO_x_ and VOC as well as O_3_. During such episodes, the nighttime NO_x_ and VOC and daytime UV levels were higher compared to non-episode days. The overall precursor levels and photochemical activity were represented fairly well by variations in the HCHO, which peaked in the morning during the high O_3_ episodes. This study revealed that toluene was the most abundant VOC in Seoul, and its concentration increased greatly with NO_x_ due to the large local influence under stagnant conditions. When O_3_ was highly elevated concurrently with PM_2.5_ under dominant westerlies, NO_x_ and VOCs were relatively lower and CO was noticeably higher than in other episodes. Additionally, the O_3_ production efficiency was the highest due to a low NO_x_ with the highest NO_z_/NO_y_ ratio among the four episodes. When westerlies were dominant in transport-south episode, the nighttime concentration of O _3_ remained as high as 40~50 ppbv due to the minimum level of NO_x_ titration. Overall, the Seoul Metropolitan Area is at NO_x_-saturated and VOC-limited conditions, which was diagnosed by indicator species and VOC/NO_x_ ratios.

## 1. Introduction

Tropospheric ozone (O_3_), which is known as a short-lived climate pollutant, is a potent greenhouse gas that acts as a product and initiator in environmental photochemical reactions (IPCC, 2007). It has been a critical cause of the increasing rate of climate change over the years and is harmful to human health, vegetation, and ecosystems due to its strong oxidation capacity (Benton et al., 2000; Fuhrer, 2003; Kampa and Castanas, 2008; Karnosky et al., 2005; Thompson, 1992; Thurston and Ito, 2001). Ozone is primarily transported from the stratosphere and produced via photochemical reactions that involve nitrogen oxides (NO_x_), volatile organic compounds (VOCs), and carbon monoxide (CO) in the presence of sunlight (NRC, 1991). These O_3_ precursors are emitted into the atmosphere from many diverse sources (e.g., vehicle exhaust, industrial activities, residences, and biogenic activities). Ozone is produced rapidly under highly elevated NO_x_ and VOC concentrations, which may lead to severe surface O_3_ pollution (Jacob, 1999). Many metropolitan areas across the globe have suffered from extreme O_3_ pollution, with severe exceedances of the National Ambient Air Quality Standards (NAAQS). For example, in North America and Europe, the number of severe O_3_ pollution events rose in the 1990s, which were alleviated by imposing strict emission controls (Chang et al., 2017; Cooper et al., 2014). Significant research has been conducted for several decades on alleviating air pollution in urban areas in Europe and North America (Jenkin and Clemitshaw, 2000; Singh et al., 2006; Solomon et al., 2000). For example, Los Angeles was troubled with photochemical smog in the 1900s; however, the O_3_ was alleviated after research was conducted and stringent control strategies were implemented on emissions (Fenger, 1999; Jacob, 1999). Similarly, Houston suffered from extreme O_3_ pollution in the summer of 2000 and the Texas Air Quality Study (TexAQS) was conducted to determine why the city frequently faced severe O_3_ exceedances of the NAAQS (Banta et al., 2005). In Mexico City, a high population density and local geographical features facilitated the O_3_ and PM_2.5_ pollution; therefore, the “Megacity Initiative: Local and Global Research Observations-the Mexico City Metropolitan Area (MILAGRO-MCMA)” campaign was conducted to improve the Mexico City Metropolitan Area emissions inventory and to understand the overall atmospheric pollution (Molina et al., 2006; Song et al., 2010).

The highest concentrations of O_3_ and other pollutants frequently occur in major metropolitan areas in South and East Asia owing to urbanization and industrialization, which is causing significant increases in O_3_ precursor emissions (Akimoto, 2003; McGranahan and Murray, 2012; Molina and Molina, 2004). In China, Beijing is one of the most populated cities and suffers from the high O_3_ pollution with severe NAAQS exceedances (Wang et al., 2006; Wang et al., 2017; Xu et al., 2011). To improve the understanding of VOC-NO_x_-O_3_ chemistry, the Campaign of Air Quality Research in Beijing and surrounding areas (CARE-Beijing) was conducted in 2006 (Chou et al., 2009).

The Seoul Metropolitan Area (SMA) accounts for approximately 44% of South Korea’s population (51 million), which has approximately 10 million registered vehicles (KOSTAT, 2019). Thus, the SMA suffers from high NO_x_ and VOC concentrations, which is a major hindrance in maintaining good air quality (An et al., 2015; Iqbal et al., 2014; Na et al., 2003). In Seoul, primary pollutants such as CO and SO_2_ decreased sharply until the early 2000s and have remained low since then (KMOE, 2017). The mean annual PM_2.5_ concentration has also decreased (Seoul, 2017). However, the O_3_ levels have clearly increased since 2005, in spite of the NO_2_ decrease. Han et al. (2013) suggested that the increased O_3_ concentration in Seoul adequately represents the complex nonlinearity between O_3_ and its precursors. In Seoul, the highest hourly O_3_ mixing ratio reached approximately 150 ppbv in the spring of 2017 (KMOE, 2017), and thus the reduction of O_3_ and its precursors has become an imminent environmental issue as well as further reduction in PM_2.5_ concentrations.

To investigate and identify the key constituents and parameters involved in the photochemical formation of O_3_, which eventually leads to O_3_ pollution, a multi-year study was conducted in the eastern parts of Seoul (at Olympic Park and Korea University) from 2004–2005 (Lee et al., 2008b; Shon et al., 2007). Moreover, the Megacity Air Pollution Study-Seoul (MAPS-Seoul) was conducted to investigate the meteorological and chemical factors that contribute to O_3_ formation in May and June of 2015 (Kim and Lee, 2018). The main result of these study was that O_3_ formation in Seoul is generally VOC-sensitive (Kim et al., 2018a; Lee et al., 2008a).

The Korea-United States Air Quality (KORUS-AQ) campaign, performed in the spring of 2016, was a comprehensive measurement study to investigate the diverse aspects of air quality problems in East Asia and to evaluate the air quality of the SMA (KORUS-AQ mission whitepaper, 2015). This study, which was conducted as part of the KORUS-AQ campaign, aims to understand the photochemical mechanisms of O_3_ formation in the SMA, diagnose its sensitivity, and identify the crucial factors that control O_3_ formation in early summer (May–June).

## 2. Experimental methods

### 2.1. Measurements

**Figure 1:**
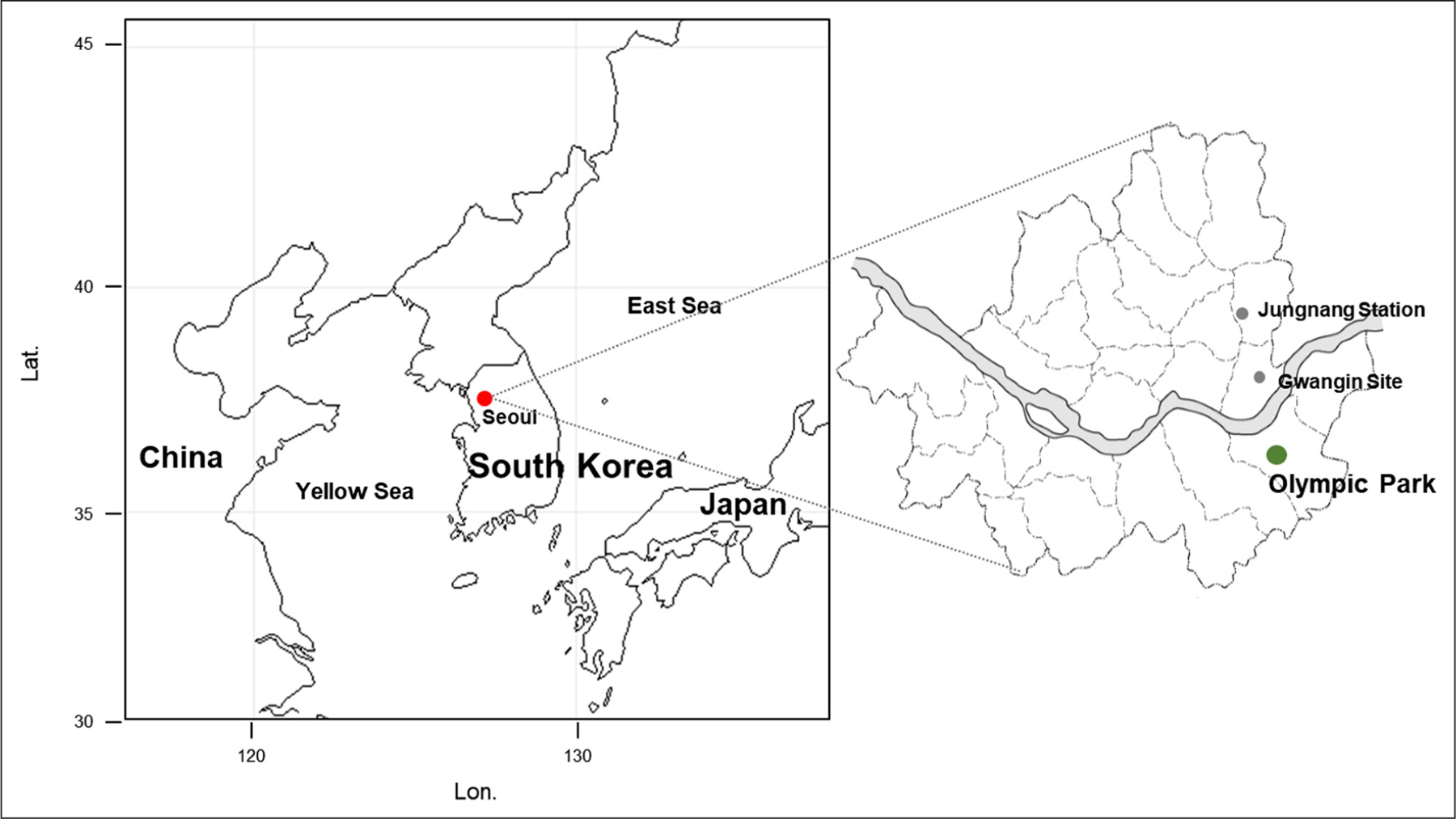
Map showing the Korean Peninsula and the capital city, Seoul. Olympic Park, utilized as a ground measurement site in Seoul, is located in the southeast portion of Seoul (37.52 N, 127.12 E). DOI: https://doi.org/10.1525/elementa.444.f1

Seoul, the capital of South Korea, is a basin surrounded by mountains with the Han River flowing across the center from east to west. The old town is located in the northern part of the Han River and forms a historical and political center, while the south is a newly developed area that serves as a residential and economic center. Ground measurements were conducted at Olympic Park, located in southeastern Seoul, from May 10 to June 12, 2016 (Figure 1). Olympic Park (37.52 N, 127.12 E) is surrounded by trees, main roads (400–600 m away), and residential areas. All measurement instruments were installed in a two-story container house, which was located close to a small lake (~50 m) and swimming stadium with a parking lot (~200 m).

During the field measurements, trace gases including O_3_, NO_x_, NO_y_, SO_2_, CO, and VOCs, aerosol species, and photochemical indicators (e.g., H_2_O_2_ and HCHO) were measured simultaneously. The Korea Research Institute of Standards and Science (KRISS) measured the O_3_, NO_x_, CO, and SO_2_ concentrations using a series of KENTEK instruments (South Korea) utilizing UV absorption (Mezus 410), chemiluminescence with a photolytic converter (Mezus 210P), non-dispersive infrared technique (Mezus 310), and UV fluorescence (Mezus 110), respectively. The detection limit was 0.5 ppbv for O_3_, NO, NO_2_, and SO_2_, and 50 ppbv for CO. The NO_x_, SO_2_, and CO instruments were calibrated every three days against zero air and span gas (400 ppbv for NO_x_ and SO_2_; 4 ppm for CO). The O_3_ monitor was calibrated before and after the field campaign using the standard reference photometer of KRISS. The NO_y_ measurement (T200U, Teledyne, USA) was made by Kunsan National University. To minimize the loss of reactive nitrogen oxide (e.g., PAN, HNO_2_, and HNO_3_) in the sample, the molybdenum converter was mounted externally, close to the sample inlet. The detection limit of NO_y_ was 0.5 ppbv. Three calibrations were performed by KRISS against NO (101.26 μmol/mol) and NO_2_ (50.05 μmol/mol) using a Teledyne (T700) calibrator. The concentration change was verified for 30 min to 1 h after injecting zero and standard gas (400 ppbv) directly through the sample line.

For VOCs, of the total 56 species of O_3_ precursors species, C6–C12 (34 species) and C2–C5 (22 species), were measured at Olympic Park and a nearby Gwangjin site (37.55 N, 127.09 E), respectively, by the Seoul Research Institute of Public Health and Environment (SRI) utilizing a gas chromatography flame ionization detector (GC-FID). In addition, the National Institute of Environmental Research (NIER) operated a proton-transfer-reaction mass spectrometer (PTR-MS) in Olympic Park to detect acetone, acetaldehyde, and methyl ethyl ketone (MEK). A detailed description of the VOC species is summarized in Kim et al., 2020.

The United States Environmental Protection Agency (EPA) installed Aerodyne quantum cascade lasers (TDL Wintel v14.91) in the second floor of container house to detect formaldehyde (HCHO). The instrument accuracy was 10%, and its precision was 0.06 ppbv (Spinei et al., 2018). A quantum cascade-tunable infrared laser differential absorption spectrometer (QC–TILDAS) was used by the Hankuk University of Foreign Studies (HUFS) to measure hydrogen peroxide (H_2_O_2_) and nitrous acid (HONO).

PM_2.5_ mass concentration and composition were measured by the SRI. The mass was monitored every 5 min utilizing beta attenuation techniques (FH 62 C14 series, Thermo Fisher Scientific). The composition was analyzed every hour for soluble ionic species using a monitoring of aerosols and gases system (MARGA, model ADI 2080, DOGA Limited, Turkey).

Meteorological parameters were monitored on site by NIER. In particular, the planetary boundary layer (PBL) height was retrieved from the continuous backscatter profiles obtained from a ceilometer (CL-51, Vaisala Inc.) by the EPA. At a nearby Jungnang site (37.5907°N 127.0794°E) that is located 8.5 km northwest of Olympic Park, vertical wind profiles were continuously monitored by pulsed Doppler wind lidar (Leosphere, Windcube-200) (Park et al., 2017). These two sets of measurements provide a unique opportunity to investigate the role of boundary layer dynamics in air quality. Considering the different time resolutions of the various measurements, all measured species were averaged hourly and merged into a dataset for further analysis in this study.

### 2.2. Model description

The air-mass trajectory was traced backward using the hybrid single-particle Lagrangian integrated trajectory (HYSPLIT) model with a global data assimilation system (GDAS, 1 degree) from the U.S. National Oceanic and Atmospheric Administration (NOAA) (Draxler and Hess, 1998; Stein et al., 2015), and the trajectories were calculated and plotted using Trajstat software (Wang, 2014).

This study used the framework for 0-D atmospheric modeling (F0AM) to simulate diurnal O_3_ variations using measured NO_x_ and VOC concentrations as hourly averages. The F0AM was initially introduced as an advanced version of 1D chemistry for the atmosphere-forest exchange (CAFE) model (Wolfe et al., 2016), but was applied to various types of experiments and analyses. The F0AM provides a chemical mechanism, such as a master chemical mechanism (MCM) or a regional atmospheric chemistry mechanism (RACM), and facilitates the simulation of an atmospheric chemistry system, including the time evolution of photochemical process, VOC oxidation, radical production, and photolysis. In this study, MCMv3.3.1, written in MATLAB, was utilized as the chemical mechanism.

## 3. Results and discussion

### 3.1. Measurement overview

**Figure 2:**
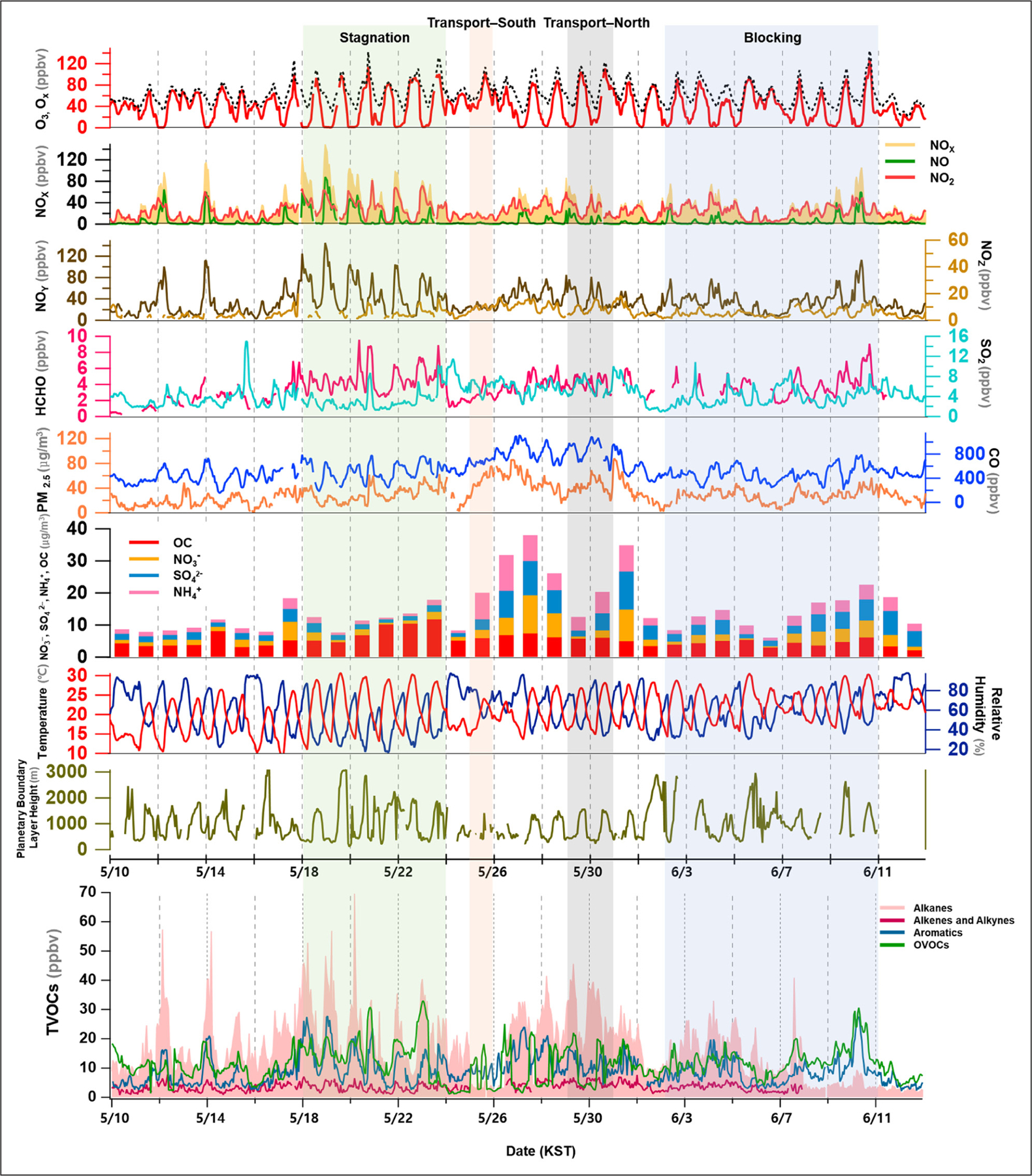
Time-series variation of reactive gases (O_3_, NO, NO_2_, NO_y_, CO, SO_2_, and HCHO), PM_2.5_ mass and composition (NO_3_^–^, SO_4_^2–^, NH_4_^+^, and OC), meteorological parameters (temperature, relative humidity, and boundary layer height), and total VOCs classified into four subgroups (alkanes, alkenes and alkynes, aromatics, and OVOCs). The C2–C5 hydrocarbons were measured at the Gwangjin site near Olympic Park. The PM_2.5_ composition is given as the daily average. DOI: https://doi.org/10.1525/elementa.444.f2

The measurement results of the trace gases (e.g., O_3_, NO_x_, NO_y_, SO_2_, HCHO, CO, and VOCs), PM_2.5_, and meteorological factors are presented for the May 10 to June 12 study period (Figure 2), and the VOCs are given for each of the sub-classes. During the entire measurement period, the daily maximum 1- and 8-h average O_3_ mixing ratios were 128 ppbv and 96 ppbv, respectively, and were observed on June 10, 2016 (Table 1). The O_3_ mixing ratios showed a variation that is typical of polluted urban sites; these variations were characterized by a clear maximum during the day and a very low level at night. The O_3_ mixing ratios, however, occasionally remained high (greater than 40 ppbv) at night with a low NO_x_. The average NO_x_ mixing ratio for the entire study period was 30 ± 23 ppbv (NO: 6.2 ± 12.1 ppbv and NO_2_: 23.9 ± 14.4 ppbv) within a range of 0.1 to 148.9 ppbv. The highest NO_x_ was observed at night on May 18. At night, the NO_x_ frequently exceeded 50 ppbv and exceeded 100 ppbv for 5 consecutive days. NO_y_ variations showed similar patterns to NO_x_ and ranged from 2.8 to 145.0 ppbv with a mean of 35.6 ± 23.3 ppbv. Furthermore, NO_z_ as [NO_y_] – [NO_x_] ranged from 1 to 20.0 ppbv, with an average of 5.9 ppbv. The total oxidant (O_x_) as the sum of O_3_ and NO_2_ exhibited a typical distribution similar to that of O_3_ with a maximum of 144 ppbv at 15:00 on June 10. In this case, O_3_ accounted for almost 90% of the O_x_ in the daytime, and the concentration of NO_2_ was mainly high during the nighttime.

As a byproduct of VOCs oxidation, HCHO was measured from May 12 to June 11, and the mean was 3.6 ± 1.6 ppbv. From May 18 to May 23, when NO_x_ was the highest, the HCHO was also elevated, and the highest HCHO was 9.6 ppbv on May 20.

During the entire experiment, the mean and maximum PM_2.5_ were 29 ± 16 μg m^–3^ and 88 μg m^–3^, respectively. The PM_2.5_ concentration exceeded the daily standard of 35 μg m^–3^ from May 25 to May 31. During this period, the major ionic species, including nitrate (NO_3_^–^), sulfate (SO_4_^2^), and ammonium (NH_4_^+^), were highly elevated and accounted for nearly one-third of the PM_2.5_ mass. In conjunction with an increase in PM_2.5_, the CO increased to 1112 ppbv, which is more than twice the average CO mixing ratio (537 ± 190 ppbv). In addition, the SO_2_ increased and remained high during this period. Unlike inorganic species, organic carbon (OC) was the most abundant from May 20–23 when PM_2.5_ concentrations were relatively low.

In this study, the four VOC sub-classes were considered as O_3_ precursors and included in the total VOCs (TVOCs): alkanes, alkenes and alkynes, aromatics, and oxygenated VOCs (OVOCs). Of these, the C2–C5 species were measured at the Gwangjin site near Olympic Park. Due to the scarcity of VOC measurements, it was not feasible to investigate the spatial homogeneity of the two sites. In Seoul, however, the OH reactivity and temporal variability of light VOCs were generally less than that of heavy VOCs (Seoul City, 2017). Therefore, the measurements from the two sites were combined for further discussion.

Among these, the alkanes were the most abundant and accounted for 40% of the total VOCs, followed by OVOCs, aromatics, and alkenes (Figure 2). The highest concentration among the individual species was acetone, followed by ethane and toluene. The highest toluene and acetone mixing ratios, 16 and 13 ppbv, respectively, occurred on June 10.

The temperature gradually increased from May to June, and the daily maximum temperature exceeded 30°C for four days during the entire period. The relative humidity varied from 16.6 to 97.8% and the PBL height significantly varied from 121 to 3,507 m according to the meteorological conditions.

### 3.2. High O_3_ episodes

#### 3.2.1. O_3_ and PM_2.5_ standard exceedance

In South Korea, the NAAQS for O_3_ are 100 and 60 ppbv for the 1- and 8-h averages, respectively. Based on these criteria, the O_3_ exceeded the NAAQS on 6 and 26 days, respectively, which means that the 8-h O_3_ standard was violated for approximately two-thirds of the experiment period. In this context, the high O_3_ episodes were selected for which the daily maximum O_3_ exceeded 90 ppbv (96^th^ percentile) in the present study. This concurred with the “moderate” phase of the Comprehensive Air-quality Index (CAI) that classifies ambient air quality according to the health risks of air pollution. As a result, in total, 14 days were chosen as high O_3_ episodes. Over the same period, PM_2.5_ exceedance occurred on 10 days, when the daily PM_2.5_ concentration was higher than 35 μg m^–3^ (1-day NAAQS for PM_2.5_).

#### 3.2.2. Synoptic weather condition

Because the study region was under the influence of the East Asian Monsoon, northerly winds were dominant from December to February, while southerly winds brought heavy rain from July to September. Before the summer monsoon season, there is a transition period during which air masses are frequently stagnant, with a low wind speed (<2 m/s), and high radiant heating during the day in May and June (Kim et al., 2018a). For the KORUS-AQ period, meteorological conditions showed dynamic variations, leading to an increase in O_3_ or PM_2.5_ concentrations (Kim et al., 2018b; RSSR, 2017). While high O_3_ events were identified under various synoptic weather conditions like the stagnant, transport, and blocking periods (Peterson et al., 2019), the PM_2.5_ exceedance days occurred mostly during the transport period.

**Figure 3:**
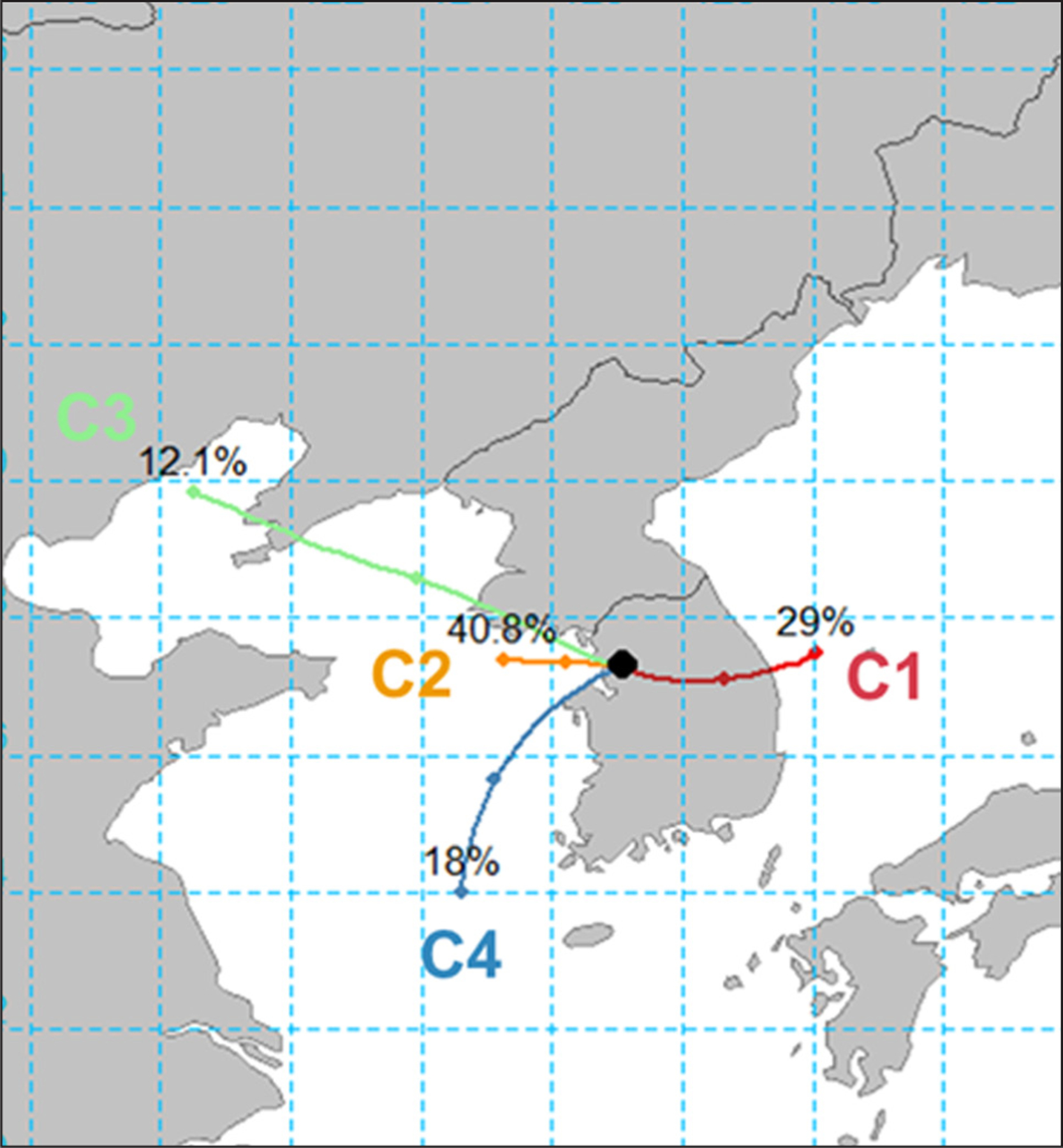
Four trajectories identified from air mass cluster analyses at 500 m for 24-h using NOAA HYSPLIT model. The high-O_3_ episode days belong to these four clusters: stagnation to C1, blocking to C2, transport-north to C3, and transport-south to C4. DOI: https://doi.org/10.1525/elementa.444.f3

With the HYSPLIT model, the 24 h backward trajectories of air masses arriving at an altitude of 500 m, were analyzed every 3 h for the entire experiment period. Through cluster analysis, these trajectories were separated into four groups (Figure 3), in which the 14 days of high O_3_ episodes were found in groups according to synoptic weather conditions. As a result, the four trajectory clusters were connected with the distinct synoptic weather periods, representing the mean trajectories of air masses for the stagnant (C1), blocking (C2), and transport (C3 and C4) periods.

**Figure 4:**
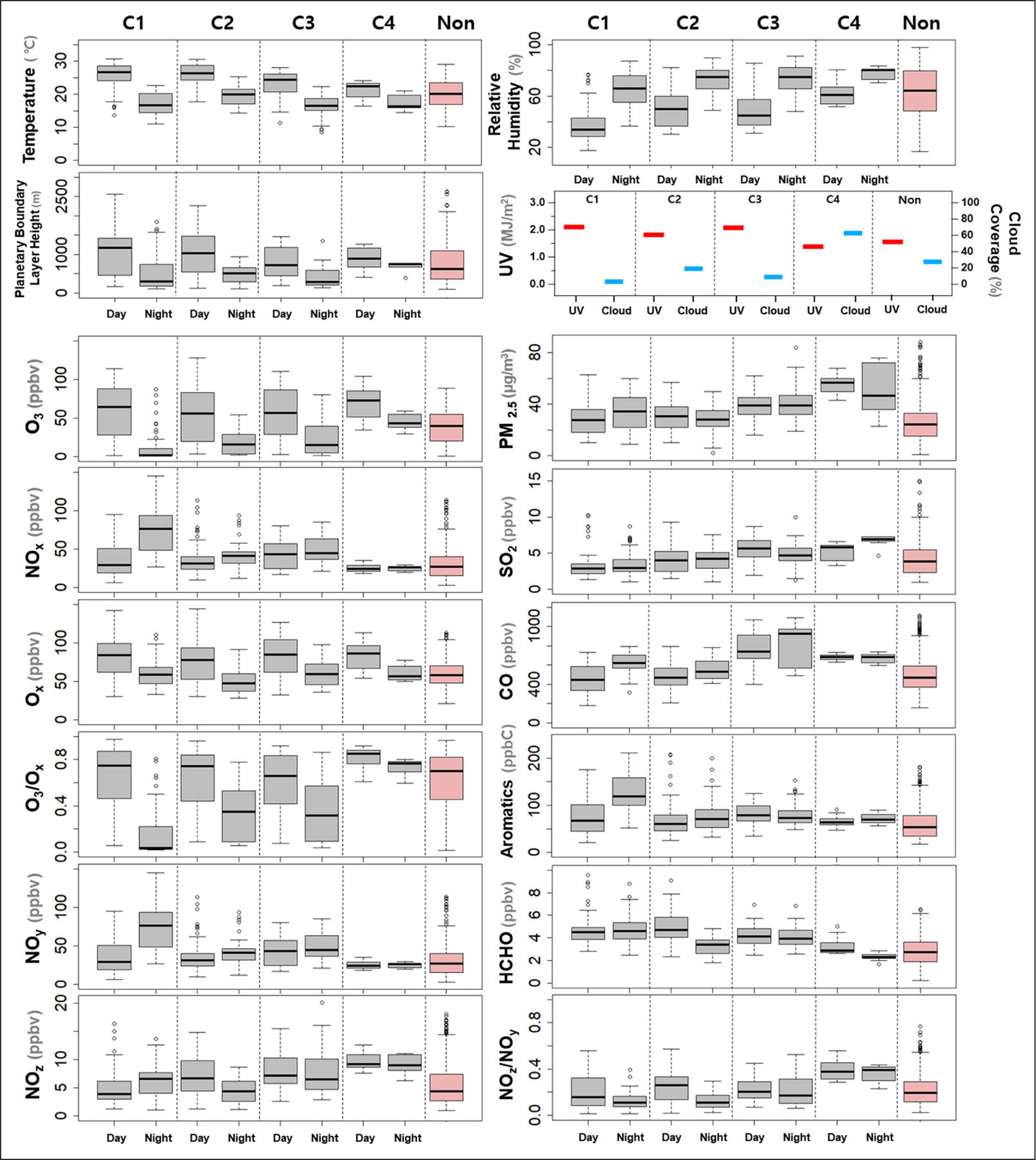
Box-whisker plots of major chemical and meteorological species and their ratios. Categorized into four high O_3_ episodes, then further divided into day (06:00–19:00) and night (20:00–05:00) and compared with the non-episode periods. UV is colored in red and cloud coverage in blue. DOI: https://doi.org/10.1525/elementa.444.f4

The episodes from May 18–23 belonged to C1 under a high-pressure system residing over the Korean Peninsula (except for May 21). Similarly, C2 included the high O_3_ events that occurred in June under a blocking pattern over East Asia (June 2, 5, 7, 9, and 10). In contrast, the episodes of C3 and C4 were characterized by highly elevated PM_2.5_ concentrations under the influence of dominant westerlies. While C3 included the impact of Northern China, including North Korea (May 17, 29, and 30), C4 was distinguished by the air mass from the southeastern part of China (May 25) (Peterson et al., 2019). Thus, the transport period was split into transport-north (C3) and transport-south (C4) period. The measurements of reactive gases and meteorological parameters were sorted by episode and compared with each other and with those of non-episode periods (Figure 4).

This study focused on the 14 days of high O_3_ episodes that were categorized into the four groups (C1–C4), for which the relevant reactive precursors were comprehensively analyzed and the factors affecting the O_3_ level were thoroughly investigated in the following section.

Temperature and relative humidity showed a gradual change from C1 to C4. Likewise, the PM_2.5_, SO_2_, and CO concentrations increased from C1 to C4. In comparison, VOC and NO_x_ were similar in variation to those of UV and PBLheight and noticeably higher in C1 than in C4. While O_3_ levels were comparable during the day, the nighttime concentration was clearly higher in C4. Among the four episodes, C1 was characterized by high temperatures, PBL heights, and NO_x_ concentrations. In contrast, C4 was distinguished by a concurrent increase in O_3_ and PM_2.5_, with high SO_2_ and low NO_x_ concentrations. The similarities and differences in the measured variables between the four episodes reveal key factors controlling the air quality and indicate the intimate coupling between chemical and meteorological processes at local and regional scales.

#### 3.2.3. Diurnal evolution of O_3_ and its precursors

**Figure 5:**
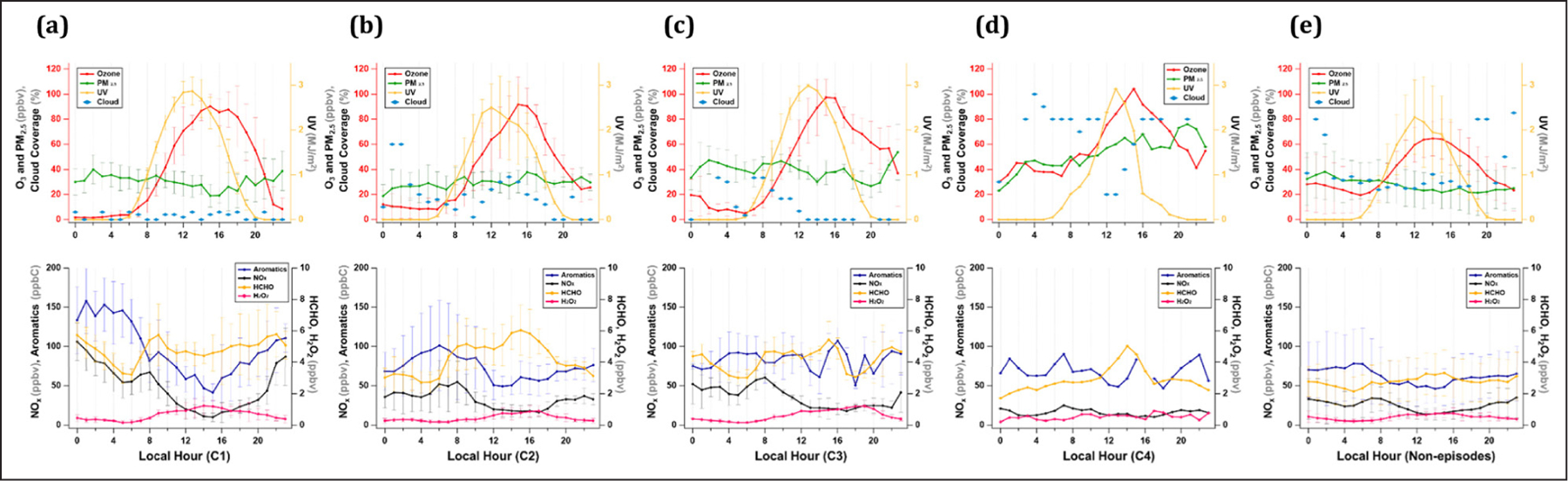
Diurnal variations of O_3_, PM_2.5_, cloud coverage, and UV (upper panel), and aromatics, NO_x_, HCHO, and H_2_O_2_ (lower panel) for four high O_3_ episodes. **(a)** C1, **(b)** C2, **(c)** C3, and **(d)** C4, and **(e)** the non-episode. DOI: https://doi.org/10.1525/elementa.444.f5

The UV and precursor levels were noticeably high during the high O_3_ episodes compared to the non-episode periods (Figure 4) and their diurnal evolution varied from episode to episode (Figure 5). In this section, therefore, the diurnal variations of the chemical species and meteorological variables were examined in detail for the four episodes. For instance, the UV level was highly variable and did not directly correlate with the O_3_ (Figure 5). In a previous study, the UV level was not a key factor that determined high O_3_ concentrations (Lee et al., 2008b). In this study, the UV level variations were related to cloud coverage (low and medium levels). In C4, the UV level was low due to a thick cloud cover in the morning, but it rapidly increased with a decrease in cloud coverage around noon, leading to a sharp O_3_ peak at 15:00 (Figure 5d). On the contrary, the O_3_ peak time was 14:00 during the non-episode period, with increased cloud coverage in the afternoon (Figure 5e). Therefore, these four high O_3_ episodes demonstrate the weather conditions that meet the prerequisites for high O_3_ formation.

One of the main results of the KORUS-AQ campaign revealed that mesoscale circulation, such as the land–sea breeze, was a critical factor determining the O_3_ level in Seoul (Peterson et al., 2019; RSSR, 2017). This was observed in the C1 episode, when stagnant condition and weak synoptic flow enhanced land-sea breeze (Peterson et al., 2019). On May 20, the O_3_ level increased abruptly and reached a maximum at 18:00.

As a major source and sink of odd-hydrogen radicals, the variations of HCHO and H_2_O_2_ were examined under different conditions during the four high O_3_ episodes and non-episode (Figure 5). Previous studies reported that HCHO reached a maximum at 10:00–11:00 or 14:00–15:00, which varied based on the season (Li et al., 2014; Pang and Mu, 2006). Most interestingly, there were three HCHO peaks identified in the present study, one in the morning, afternoon, and evening. The morning peak appeared right after the maximum NO_x_ occurred and was most pronounced in C1 when there was no typical afternoon maximum for HCHO (Figure 5a). Instead, the HCHO increased with NO_x_ at night, which was the most noticeable on May 21 and 22 (Figure 2). This is primarily related to the daily evolution of boundary layer.

In the stagnant condition (C1), NO_x_ and VOC levels were highly elevated at night, but at a minimum in the afternoon. While the shallow PBL resulted in the enrichment of precursors at night, all precursor levels were at their minimum levels when the boundary layer was the deepest in the afternoon. In the morning, the UV level was the highest for all four episodes, which likely expedited VOC oxidation and led to HCHO formation, and in turn, HO_2_ radical production. It was recently hypothesized that OH radicals produced from HONO photolysis initiated VOC oxidation early in the morning during the high O_3_ episodes (Gil et al., 2020). It is likely to be a plausible reason for the morning HCHO peaks observed in this study.

In general, the afternoon peak was evident and represented the daily maximum HCHO, which was the most pronounced on May 25 (C4) (Figure 5d). The daily maximum was not significantly different between episodes (5–6 ppbv), although it was substantially lower during non-episodes. Likewise, the background HCHO level was higher during the high O_3_ episodes (~4 ppbv) than during non-episodes (~3 ppbv). Furthermore, the morning and night HCHO peaks agreed well with the VOC and NO_x_ levels. These results imply that HCHO serves as a robust tracer for the overall VOC activity that leads to O_3_ formation. The minimum HCHO for the entire measurement period, which was no less than 2 ppbv, indicates that it needs to be further investigated to determine whether it was a result of primary or secondary sources.

In the four episodes, the diurnal variation of H_2_O_2_ was different in the peak time between 14:00 and 19:00. Except for C1, H_2_O_2_ reached its maximum around 17:00, after the O_3_ peak. In C1, the H_2_O_2_ mixing ratio increased in the early morning and showed a broad maximum around 14:00, before the O_3_ maximum (Figure 5a). This is likely associated with the morning HCHO peak, which readily produced HO_2_ radicals and promoted HO_2_ formation. In the present study, the H_2_O_2_ levels were higher during the high O_3_ episodes than during the non-episodes; however, the maximum barely exceeded 1 ppbv due to the high NO_x_, which was particularly high at night. In C1, the boundary layer was the most intensively expanded during the day, and the levels of primary species rapidly decreased accordingly, creating a favorable condition for H_2_O_2_ formation.

These findings demonstrate that the photochemical characteristics differed between episodes and that the cycle of odd-hydrogen radicals was closely related to the overall precursor level and meteorological conditions.

### 3.3. Planetary boundary layer effects on air quality

**Figure 6:**
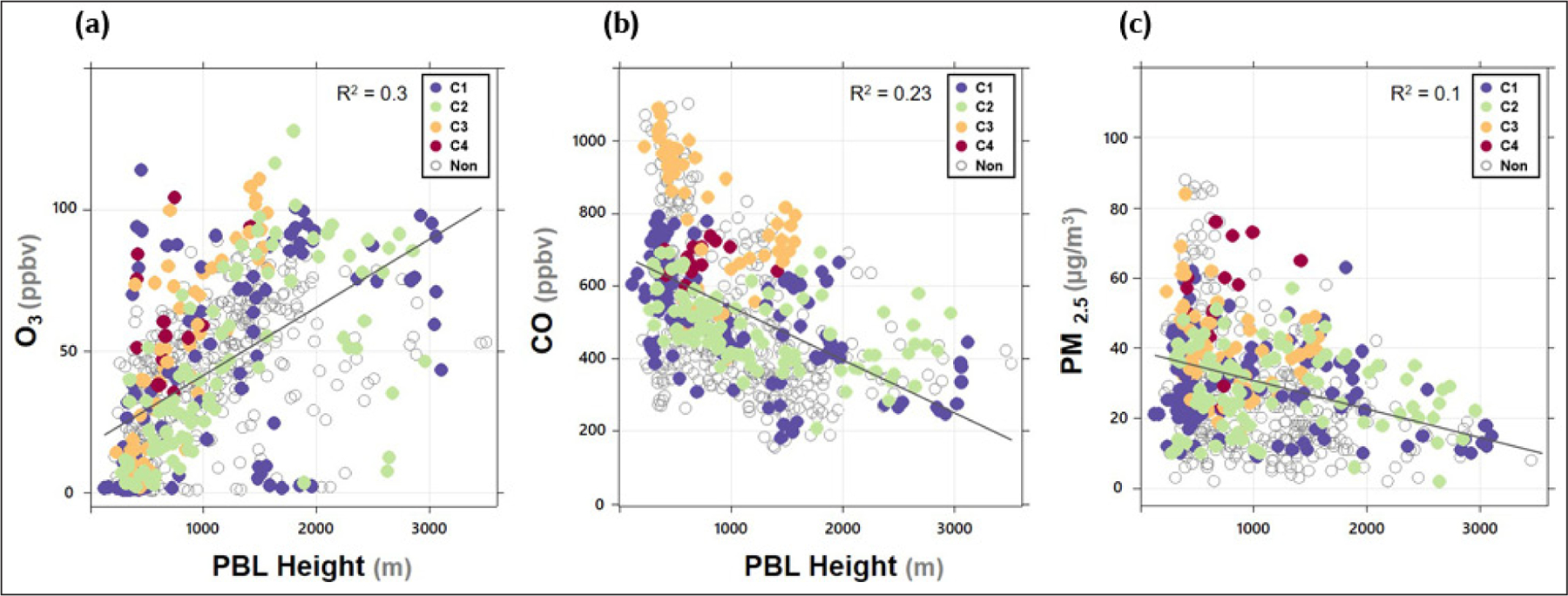
Correlation between planetary boundary layer (PBL) height and (a) O_3_, (b) CO, and (c) PM_2.5_ for the high O_3_ episodes and a non-episode. DOI: https://doi.org/10.1525/elementa.444.f6

In Northeast Asia, atmospheric pollutant levels are significantly affected by synoptic weather conditions, which was also observed in this study (Jordan et al., 2020; Peterson et al., 2019). Additionally, the daily evolution of the boundary layer has been found to be a critical factor determining urban air quality (e.g., Huang et al., 2018). As expected, in the present study, O_3_ and CO were positively and negatively correlated with the PBL height, respectively (Figure 6). The measurement data presented for each episode shows that the boundary layer was deeper in C1 and C2 than in C3 and C4. Nevertheless, O_3_ levels were comparable for the four episodes because a deep boundary layer not only promotes O_3_ production through NO_x_ dilution, but also dilutes photochemically produced O_3_. In Figure 6a, outliers were mostly observed in C1 and C2. Especially, on May 20 (C1), the O_3_ concentration rapidly increased with a decrease in the PBL height, reaching up to 113 ppbv at 18:00. It turned out that this was caused by mesoscale circulation, such as a land–sea breeze (Peterson et al., 2019).

The relationship between PBL height and CO demonstrates that the CO level was affected by the PBL because it was diluted during the day and accumulated at night (Figure 6b) and was evidently higher during transport episodes (C3 and C4) than the other episodes. This characteristic was partially attributed to the change in the PBL height under different synoptic meteorological conditions. Like CO, PM_2.5_ concentration was inversely related to the PBL height (Figure 6c). Considering that the highest PM_2.5_ was due to secondary inorganic ions at night in C4 episode, this relationship suggests that there must be other mechanisms responsible for nighttime increases in PM_2.5_ than accumulation or transport, which is addressed in Jordan et al. (2020).

**Figure 7:**
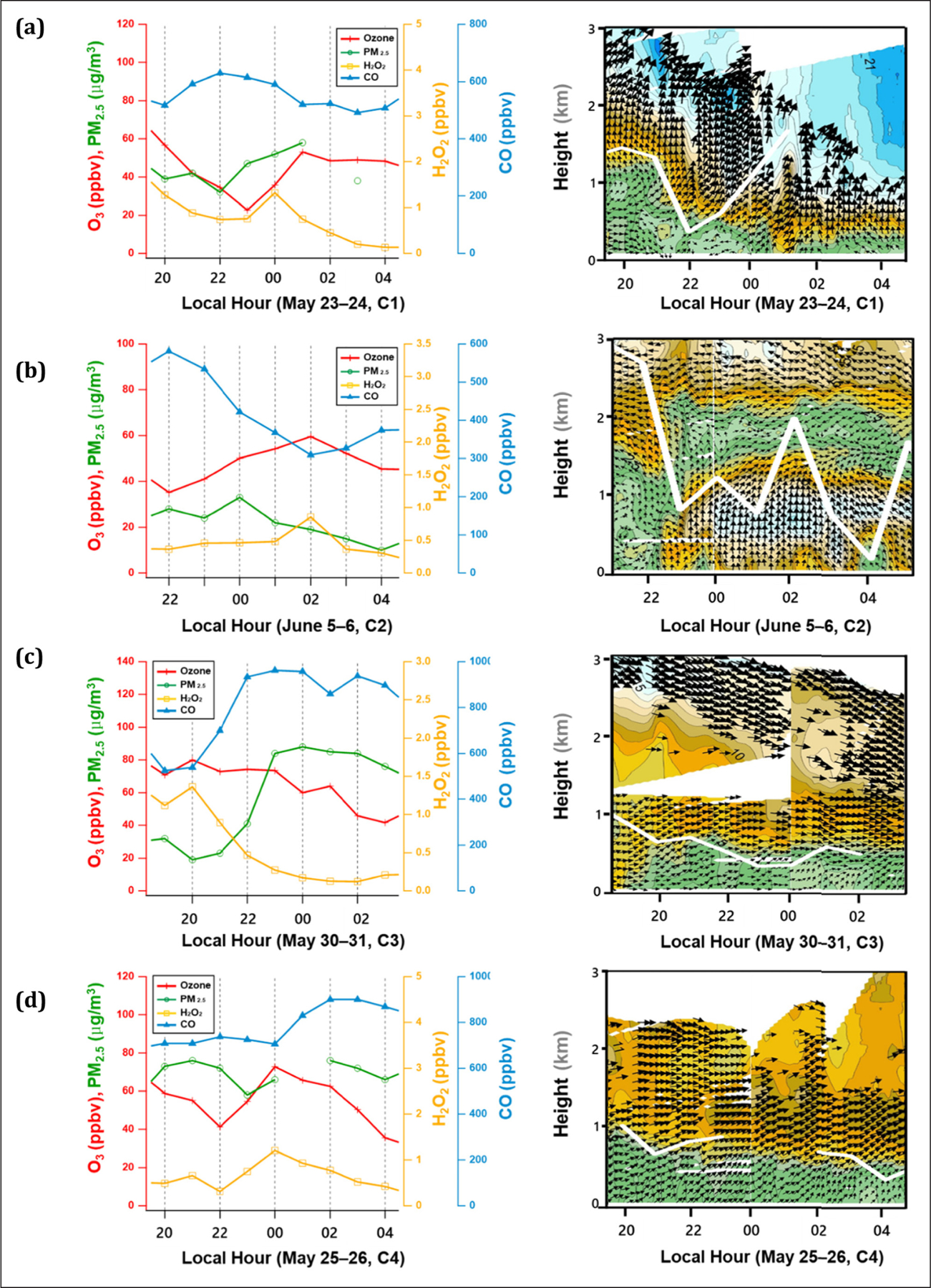
Diurnal variations of O_3_, PM_2.5_, CO, and H_2_O_2_ at night with the boundary layer height (white line) and vertical wind profile (wind speed and direction) up to 3 km measured by a pulsed Doppler wind lidar at the Jungnang site near Olympic Park on (a) May 23–24, (b) June 5–6, (c) May 30–31, and (d) May 25–26. DOI: https://doi.org/10.1525/elementa.444.f7

In addition to the high O_3_ occurrence observed on May 20, O_3_ often increased or remained high after sunset, which was associated with the low PBL height as shown in Figure 6a. On May 23–24 and June 5–6, the O_3_ increase was accompanied with an increase in H_2_O_2_ and a decrease in CO from 00:00–02:00 (Figure 7a and **b**). These events took place in association with a rapid change in the PBL height (C1 and C2, respectively). In comparison, the nighttime O_3_ and H_2_O_2_ enhancement was concurrent with an increase in CO and PM_2.5_ on May 25–26 and 30–31, during which the PBL height remained low (C3 and C4, respectively) (Figure 7c and **d**). The PBL was overlaid with the vertical wind vectors measured by the Doppler wind lidar in Figure 7, which revealed the detailed boundary layer structure, particularly when the PBL was shallow at night.

For cases C1 and C2, the PBL change was associated with strong southerly winds above the boundary layer and at the surface, respectively. These horizontal winds were possibly due to local circulation such as land and mountain breezes, which caused the boundary layer to become unstable and the nocturnal residual layer was mixed down. The PBL primarily stayed shallow under constant westerlies; however, it was slightly increased when winds were strong, as indicated in C4 (Figure 7d). In this case, CO and PM_2.5_ concentrations increased with O_3_ and H_2_O_2_ increases. As a result of the KORUS-AQ campaign, the elevated CO/CO_2_ ratio was suggested to be an indicator of Chinese influences (Tang et al., 2018). It is mainly attributed to the CO enhancement, which is evident in the transport regimes of C3 and C4.

Given that all these enhanced O_3_ events at night occurred during the high O_3_ episodes, the likely cause is the vertical mixing of air enriched with photochemical byproducts in the residual layer. In previous studies, the nighttime O_3_ increase has been explained by nocturnal residual layer entrainment (Aneja et al., 2000; Jackson and Hewitt, 1996; Lee et al., 2008b). Therefore, the results of this study demonstrated that, in addition to synoptic circulation, the daily boundary layer evolution was responsible for the O_3_ and PM_2.5_ short-term variations as well as primary pollutants such as CO. The enhanced nighttime O_3_ level was not as high as the episodes during the day; however, it was greater than 60 ppbv and could thus contribute to the 8-h NAAQS violations. It is also note-worthy that the nighttime increase was accompanied by an increase in H_2_O_2_; thereby suggesting that it is a useful tracer for residual layer entrainment.

### 3.4. Characteristics of O_3_ precursors

#### 3.4.1. Nitrogen oxide

In general, O_3_ is titrated by NO_x_ under a high NO_x_ environment; this is a major process of O_3_ loss in urban areas. Thus, O_x_ (NO_2_+O_3_) represents the actual O_3_ level and is a better indicator for understanding O_3_ chemistry (Lei et al., 2007; Lin et al., 2008). As shown in Figure 4, the O_x_ level was comparable in all four cases. For O3, the daytime levels were in a similar range, but the nighttime levels were clearly different from episode to episode, leading to a large difference in the O_3_/O_x_ ratio at night caused by a difference in NO_x_, which were highest in C1 and lowest in C4. The difference of nighttime O_3_/O_x_ ratios in the C1 and C4 demonstrates that the NO_x_-titration effect is significant even during the high O_3_ episodes in Seoul. It is also note-worthy that the nighttime O_3_ concentration remained as high as 40–50 ppbv under the low NO_x_ condition in C4, which is comparable to the mode concentration of O_3_ (59 ppbv) observed from shipboard measurements in the Yellow Sea during the KORUS-AQ (Seo et al., 2019).

Like NO_x_, the NO_y_ mixing ratio was two times higher in C1 (52.5 ppbv) than in C4 (24.8 ppbv). In contrast, NO_z_, calculated as [NO_y_] – [NO_x_], was the highest in C4, in which NO_z_ accounted for approximately 40% of the NO_y_. It is well known that the NO_z_/NO_y_ ratio provides information about the photochemical aging of air masses (Marion et al., 2001; Perros and Marion, 1999; Wood et al., 2009). The NO_z_/NO_y_ ratio was higher in C3 and C4 than in C1 and C2, as expected from the meteorological regime of the air mass. NO_z_ is discussed further in Section 3.5.2 to evaluate O_3_ production efficiency.

#### 3.4.2. Volatile organic compounds

**Figure 8:**
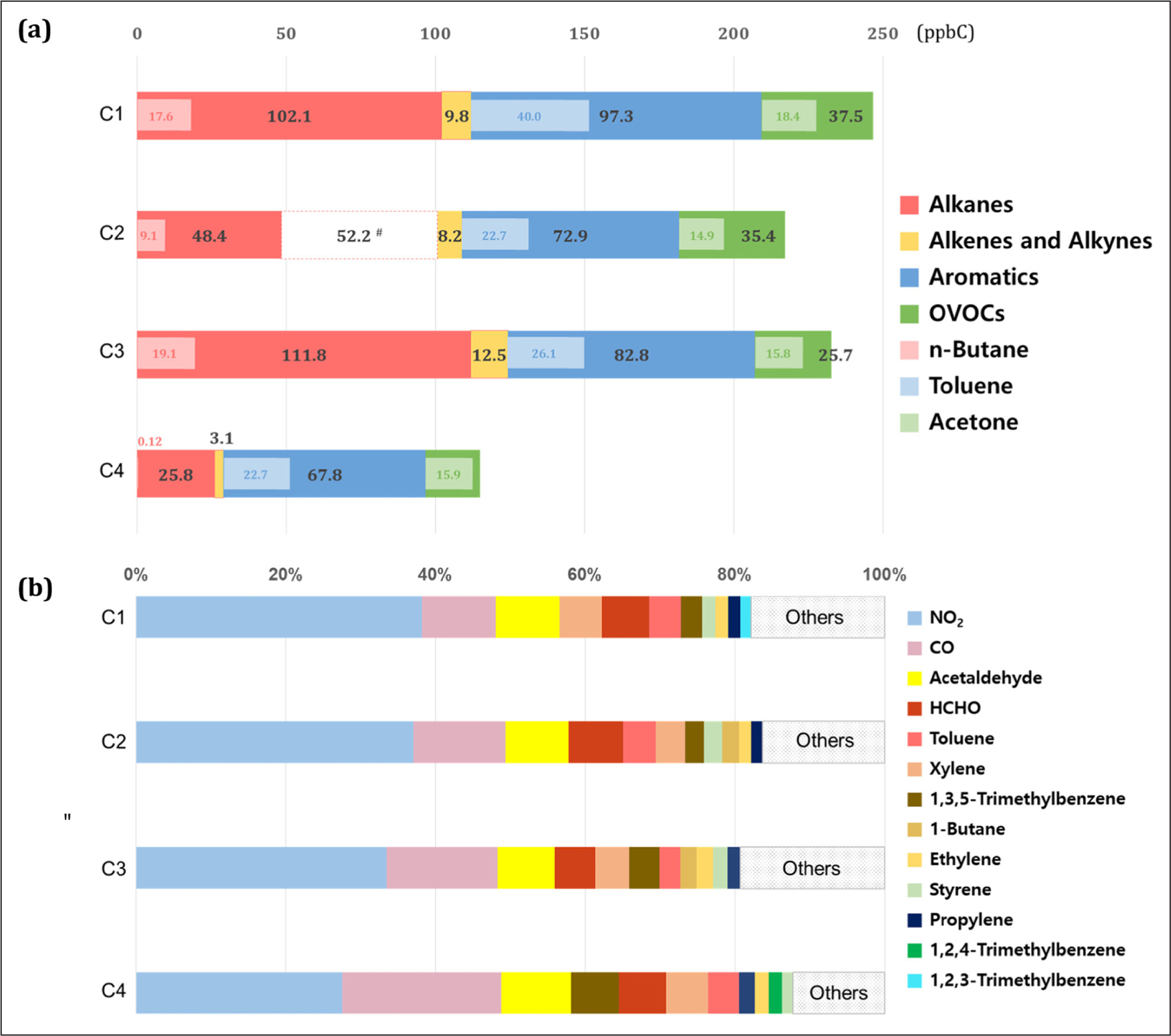
(a) Abundance of the four VOC sub-classes and individual VOC species and (b) their relative OH reactivity with that of NO_2_ and CO during the four high O_3_ episodes. For this analysis, the missing C2–C5 alkane measurements during June 8–12 were estimated from a linear regression between alkanes and aromatics (R^2^ = 0.65) and are presented separately with a red dashed line. DOI: https://doi.org/10.1525/elementa.444.f8

The reactions of NO with HO_2_ and RO_2_ radicals produced from VOC oxidation are the major pathway for radical cycling that leads to O_3_ formation. In general, VOC reactivity varies over a wide range and individual VOC species make different contributions to O_3_ formation in the atmosphere (Atkinson et al., 2000). The TVOC mixing ratios were higher during the high O_3_ episodes (203.0 ppbC) than during the non-episodes (154.7 ppbC). Among the four episodes, TVOCs were the highest in C1, followed by C3, and the lowest in C4 (Figure 8a). Toluene was the most abundant VOC compound, accounting for nearly 15% of the TVOCs, followed by acetone and n-butane. It was the highest in C1 among the four episodes.

To compare the contribution of each VOC species to O_3_ formation, their OH reactivity was calculated (Atkinson et al., 2003) including NO_2_ and CO (Figure 8b). Although alkanes were abundant in Seoul, they were less reactive than the aromatics such as xylene and toluene or OVOCs such as acetaldehyde and HCHO. These aromatic VOCs were found to significantly influence O_3_ production during the KORUS-AQ (Schroeder, 2020). Despite their low mixing ratios, 1,3,5-trimethylbenzene and styrene exhibited a considerably high contribution to OH reactivity.

The OH reactivity of CO and NO_2_ comprised approximately half of the total reactivity for all episodes. Interestingly, the CO contribution increased with the decrease in NO_2_ reactivity from the C1 to C4 episodes. CO has not been regarded as a major O_3_ precursor in urban chemistry due to its low reactivity and long lifetime (cf., Jacobson, 2002; Sze, 1977); however, its contribution was reported to be significant in previous studies (Di Carlo et al., 2004; Jeffries, 1995; Vukovich, 2011) and increased under a transport regime in this study. Since the results of this study are based entirely on measurements, the CO contribution to the total OH reactivity may be over-estimated due to missing OH reactivity or VOC measurement uncertainty. Basically, the increased contribution of OH reactivity was due to the elevated CO under transport regime in the C3 and C4 episodes, during which the average OH reactivity of CO was 3.1 and 2.8 s^–1^, respectively, compared to the average OH reactivity of CO was 2.1 s^–1^ for the entire period. This analysis shows that the photochemical properties vary depending on weather conditions, which may have implications for policy making.

As the most abundant carbonyl compounds in an urban area, formaldehyde and acetaldehyde significantly contributed to OH reactivity (15%) during the high O_3_ episodes. These compounds are mainly produced by secondary formations from the breakdown of VOCs or directly emitted from a variety of sources (Luecken et al., 2012; Ling et al., 2017). In previous studies, their importance to O_3_ formation was demonstrated in many regions, including the SMA (Shao et al., 2009; Kim et al., 2015).

### 3.5. O_3_ formation diagnosis

#### 3.5.1. Ozone formation sensitivity

O_3_ formation sensitivity to NO_x_ or VOCs is driven by odd-hydrogen chemistry, which can be diagnosed by photochemical indicator species such as H_2_O_2_, HNO_3_, HCHO, and NO_z_ (Milford et al., 1994; Sillman, 1995; Sillman et al., 1997). In models, NO_x_- and VOC-sensitive regimes were associated with the high and low values of H_2_O_2_/HNO_3_, O_3_/NO_z_, and O_3_/NO_y_ ratios. In the present study, the evidence for O_3_ formation sensitivity was obtained from NOy, NOz, HCHO, and H2O2 measurements.

The split values of various indicators (Sillman, 1995; Sillman et al., 1997) and the percentage of measurements that fit into these criteria are summarized in Table 2. For the NO_y_ indicator, 63% of the measurements fell into the VOC-sensitive regime. When the H_2_O_2_/NO_y_ ratio was applied, 96% of all the measurements fell into the VOC-sensitive regime. All the other indicator species ratios tested in this study, including O_3_/NO_y_, H_2_O_2_/NO_z_, and HCHO/NO_y_, consistently indicated that O_3_ formation is more sensitive to the VOC than the NO_x_ in Seoul (Table 2). This result demonstrates that VOCs play a more critical role than NO_x_ in O_3_ production, thereby suggesting VOC control for O_3_ abatement.

**Figure 9:**
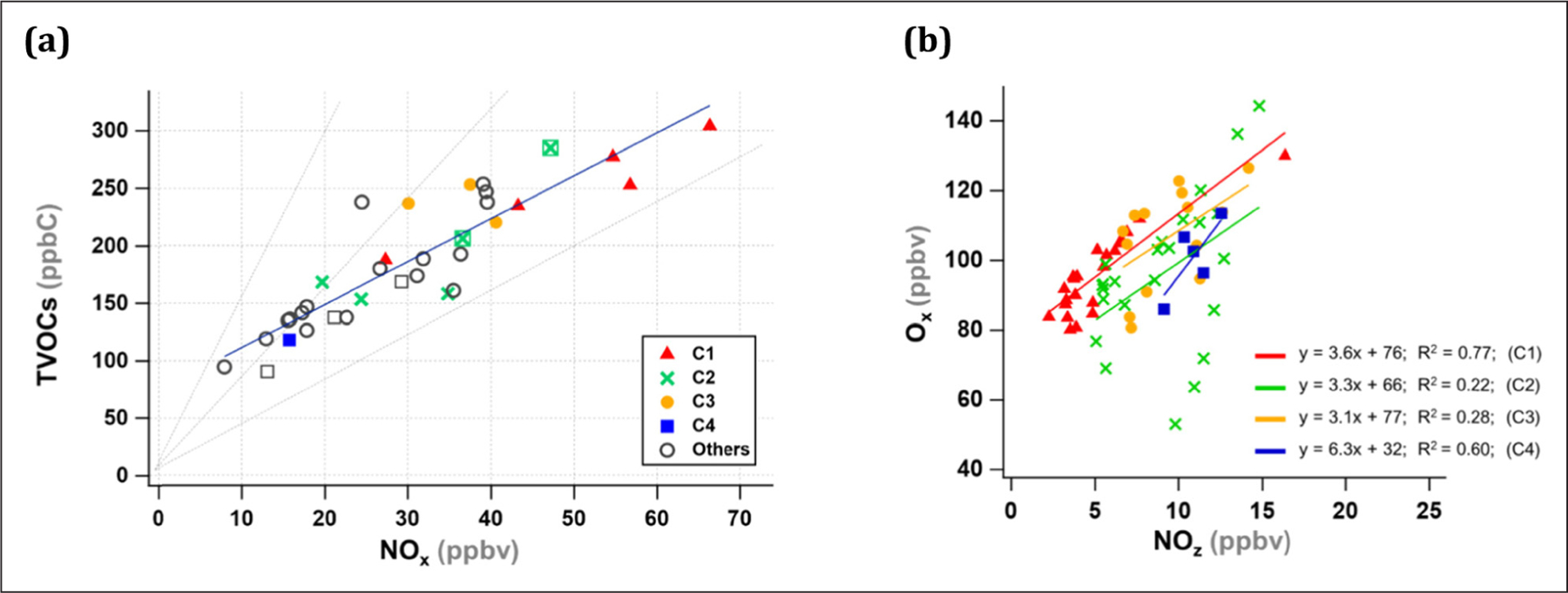
(a) Daily averaged TVOCs and NO_x_ concentrations and (b) correlation of NO_z_ with O_x_ between 12:00 and 16:00, signifying OPE during the four high O_3_ episodes. In (a), the line denotes the TVOCs/NO_x_ ratio of 15, 8, and 4. The estimated TVOCs (see Figure 8) are presented as open squares for June 8–12. DOI: https://doi.org/10.1525/elementa.444.f9

Before this approach was developed, the relative ratio of VOCs to NO_x_ was utilized as a diagnostics tool to evaluate the O_3_ production regime (United States Environmental Protection Agency, 1989). The TVOCs-to-NO_x_ ratios greater than 15 and less than 4 are often considered as NO_x_- and VOC-sensitive regimes, respectively (NRC, 1991). Thus, this method was applied to this study, and the daily averaged VOC and NO_x_ mixing ratios were obtained as shown in Figure 9a. Consistent with the results presented earlier, the O_3_ formation was found to be VOC-limited and 13 of the 14 high O_3_ days fell into the line of TVOCs: NO_x_ between 4 and 8. In comparison, a few non-episode days were found in the transition regime. The indicator species ratio and the relative ratio of TVOCs to NO_x_ consistently indicated that O_3_ formation is NO_x_-saturated and VOC-limited in Seoul, and therefore VOCs need to be reduced to decrease the O_3_ concentration. It is also noteworthy that although the TVOCs/NO_x_ ratios are similar for high O_3_ episodes, the individual NO_x_ and VOC mixing ratios were spread in a wide range and are not distinguished from those of non-episode days, except for C1. In this regard, the detailed chemical mechanisms tightly linked with meteorological conditions of the high O_3_ episodes should be thoroughly understood when establishing an O_3_ abatement policy.

#### 3.5.2. Ozone production efficiency

In addition to the sensitivity regime, ozone production efficiency (OPE) is another key factor in determining O_3_ mixing ratio in the NO_x_-saturated regime (Kleinman et al., 2002a; Zaveri, 2003). OPE can be practically determined from a linear regression of O_3_ or O_x_ against NO_z_ (Kleinman et al. 2002b; Trainer et al., 1993). In previous studies, OPE has been found to be as high as 10 in low NO_x_ conditions (Li et al., 1997; Trainer et al., 1993). In urban conditions, low OPEs were found as follows: 8 in Houston during the DISCOVER-AQ campaign (Mazzuca et al., 2016), 5.9 in Texas during the TexAQS 2006 field study (Neuman et al., 2009), 5.4 during the TexAQS 2000 (Ryerson et al., 2003), 2.2–4.2 in New York City (Kleinman et al., 2000), and 6 and 5 for urban and power plant plumes in Nashville, respectively (St John et al., 1998).

In this study, OPE was determined as a slope of O_x_ against NO_z_ between 12:00 and 16:00. For each high O_3_ episode, it ranged from 3.1 to 6.3 mol/mol (Figure 9b). As expected, it was the highest in C4 (6.3 mol/mol), which was twice as high as the other episodes (~3 mol/mol), suggesting that O_3_ production was more efficient under the transport regime. In the C4 episode, NO_x_ and VOC levels were significantly lower; however, the NO_z_ levels and the NO_z_/NO_y_ ratios were considerably higher than in the other episodes (Figure 4). The O_x_ to NO_z_ ratio also serves as an indicator for O_3_ formation sensitivity and is higher under NO_x_-sensitive and lower under VOC-sensitive regimes (Kleinman et al. 2002b; Sillman et al., 1995). This result implies that the NO_x_ titration effect is substantial in Seoul and reducing NO_x_ will increase OPE and thus O_3_. Currently, high PM_2.5_ concentrations have become a national issue, and the NO_x_ reduction policy has been implemented to combat PM_2.5_. In this respect, an integrated understanding of PM_2.5_ and O_3_ formation is required when establishing policies to improve urban air quality.

### 3.6. Effect of NO_x_ and VOC reduction on O_3_

In the present study, the precursor levels were higher in the high O_3_ episodes than the non-episodes and the O_3_ formation was controlled by VOCs during high O_3_ episodes. In this study, the FOAM modeling was utilized to examine how O_3_ level changed as precursor levels were reduced to non-episode levels. Evaluating the NO_x_ and VOC reduction effect on O_3_ production is crucial for implementing O_3_ policies. First, the O_3_ mixing ratio was simulated for episodes C1, C2, and C3 with the average diurnal profiles of NO_x_ and VOCs. In this simulation, C4 was not considered because the NO_x_ and VOC levels were lower than during the non-episodes. Next, O_3_ was calculated with the three reduced scenarios of NO_x_, VOCs, and combined NO_x_ and VOC. Then, the peak O_3_ level of each run was compared (Table 3). The detailed photochemical model information used for this calculation can be found in Gil et al., 2020. Considering the abundance and OH reactivity, ten VOC species were chosen for simulation including acetaldehyde, acetone, BTX, and isoprene. These species account for nearly two-thirds of the total OH reactivity of the 56 VOC species.

For the C1 episode, NO_x_ and VOC levels were reduced by 49% and 45%, respectively, compared to the base run. For C2 and C3, the NO_x_ and VOC reductions were 24% and 16%, and 31% and 34%, respectively. As expected from the O_3_ formation sensitivity, O_3_ increased with a reduction in NO_x_ but decreased with a reduction in VOCs in all three episodes. While the increased O_3_ was not related to the NO_x_ reduction, the O_3_ decrease was proportional to the VOC reduction. When reducing both NO_x_ and VOCs, the decrease in O_3_ was less than the VOC-only reduction. This model simulation highlights that the chemical regime creating high O_3_ mixing ratio was highly saturated with NO_x_ and the daily maximum O_3_ was reduced by 0.57% per 1% reduction in VOCs regardless of the NO_x_ level and VOC composition.

## 4. Conclusion

As a part of the KORUS-AQ campaign, comprehensive measurements were conducted at several ground sites in South Korea. At Olympic Park in Seoul, reactive gases including O_3_, NO_x_, NO_y_, CO, and SO_2_, and VOCs, photochemical byproducts such as HCHO and H_2_O_2_, PM_2.5_ composition, and meteorological parameters such as PBL height were continuously measured from May 10 to June 12, 2016. In this experiment, the maximum hourly O_3_ (127 ppbv) and PM_2.5_ (88 μg m^–3^) occurred on June 10 and May 31, respectively. The average NO_x_ and TVOC were 30.0 and 39.3 ppbv, respectively. As the most dominant VOCs, the toluene mixing ratio reached 16.0 ppbv with an average of 3.9 ppbv.

During the experiment, the O_3_ level violated the 8-h standard of 60 ppbv for 26 days and the 1-h standard of 100 ppbv for 6 days. For an in-depth analysis of this study, the high O_3_ episode was defined as occurring when the daily O_3_ maximum exceeded 90 ppbv (96^th^ percentile). The 14 days of high O_3_ episodes occurred under distinct weather conditions that were separated into stagnant, transport, and blocking periods. The transport regime was further divided into two periods involving different air masses from the southwest (transport-south) and northwest (transport-north). As the experiment was conducted before the summer monsoon started, the synoptic meteorology played a significant role in determining the air quality. For example, PM_2.5_ and O_3_ were imported in aged plumes transported over the Yellow Sea under the constant westerlies and the local influences were more significant as westerlies were weakened. When the extreme stagnation developed under a persistent anticyclone, the evolution of boundary layer played an important role.

Under a persistent high pressure, NO_x_ and VOC were greatly elevated, particularly at night by the emissions of the SMA. During mornings when the UV level was high under a clear sky, the VOC oxidation resulted from a clear morning peak in HCHO. In the afternoon, all precursor levels decreased below those of the non-episode days due to deep mixing. During stagnant periods, O_3_ occasionally increased in the evening and at night, which was associated with mesoscale circulation and was evident in the vertical wind profile.

Similarly, the air was stagnated under blocking condition. In this condition, the precursor levels were lower than in the stagnant episode and the levels and variations of photochemical byproducts highlight that the air was photochemically more aged with higher HCHO and NO_z_ levels during the day. Accordingly, the highest O_3_ concentration was observed in this episode.

When the air mass was transported across the Yellow Sea, O_3_ was concurrently enhanced with PM_2.5_. This episode is characterized by high CO and SO_2_ levels, but low NO_x_ and VOC concentrations. As a result, O_3_/O_x_ and NO_z_/NO_y_ ratios were high, implying that the air was photochemically aged. At night, O_3_ concentrations remained in the range of 40–50 ppbv with an O_3_/O_x_ ratio as high as 0.8, indicating the O_3_ titration by NO_x_ is substantial and played a critical role in determining the O_3_ level in Seoul. In addition, the OH reactivity of CO was increased with a decrease in NO_2_. CO was highly enhanced with O_3_ under the northerly wind at the end of May.

For the high O_3_ episodes, the O_3_ formation was diagnosed as VOC-sensitive from the photochemical indicator species ratios such as H_2_O_2_, HCHO, and NO_z_, and from the TVOC and NO_x_ levels. In addition, the ozone production efficiency evaluated from the ΔO_x_/ΔNO_z_ of the linear regression between O_x_ and NO_z_ was higher in the transport regime than in the other episodes, which was further demonstrated from the model simulation. Because the precursor levels were higher during the high O_3_ episodes than during the non-episodes, the diurnal variation of O_3_ was simulated for the three episodes (C1–C3) with the NO_x_ and VOC levels of the non-episode. Under the NO_x_-saturated condition, the NO_x_ reduction increased the O_3_ concentration. In contrast, the daily maximum O_3_ was reduced proportionally with the VOC reduction rate; therefore, reducing NO_x_ would exacerbate air quality in terms of O_3_. In the SMA, air quality improvement should be attained, based on a comprehensive understanding of the O_3_ and PM_2.5_ formation.

## Supplementary Material

Sup Data 3

Sup Data 2

Sup Data 1

## Figures and Tables

**Table 1:** Measurement statistics of O_3_, PM_2.5_, NO_x_, NO_y_, CO, SO_2_, VOCs, HCHO, and meteorological parameters during the KORUS-AQ campaign, and the number of days (out of total 34 days) during which O_3_ and PM_2.5_ values exceeded the NAAQS. DOI: https://doi.org/10.1525/elementa.444.t1

Species	Mean ± SD (ppbv)	Maximum (ppbv)	Exceedance days

O_3_	39 ± 27	128	6^a^, 26^b^
PM_2.5_	29 ± 16 (µg m^−3^)	88 (μg m^−3^)	23^c^
NO_x_	30 ± 23	149	–
NO_y_	36 ± 23	145	–
CO	537 ± 190	1113	–
SO_2_	4.2 ± 2.1	15.0	–
TVOCs	39 ± 18	105	–
Alkanes	16 ± 11	69	–
Alkenes and Alkynes	4 ± 1	8	–
Aromatics	9 ± 5	27	–
OVOCs	10 ± 3	26	–
Formaldehyde	3.6 ± 1.6	9.6	–

Temperature (°C)	20.8 ± 4.8	30.6	–
Relative Humidity (%)	60.7 ± 19.4	97.8	–
Planetary Boundary Layer Height (m)	774 ± 513	2624	–
Wind Speed (m/s)	0.5 ± 0.4	2.3	–

a Exceedance of 1-h standard of 100 ppbv.

b Exceedance of 8-h standard of 60 ppbv.

c Exceedance of 24-h standard of 35 μg m^−3^.

**Table 2:** Percentage of measurements that meet the criteria of each indicator for VOC- and NO_x_-sensitive regimes. DOI: https://doi.org/10.1525/elementa.444.t2

Indicators	VOC-sensitive	NO_x_-sensitive

NO_y_	63% (> 20 ppbv)	11% (< 10 ppbv)
O_3_/NO_y_	85% (< 6)	9% (> 7.5)
H_2_O_2_/NO_y_	96% (< 0.15)	0% (> 0.3)
H_2_O_2_/NO_z_	75% (< 0.2)	9% (> 0.35)
HCHO/NO_y_	69% (< 0.2)	4% (> 0.4)

**Table 3:** Average concentration of O_3_, PM_2.5_, NO_x_, and TVOCs during the four high O_3_ episodes, as well as the non-episode periods, and the rate of change in O_3_ maximum concentration with a reduction in NO_x_, VOCs, and combination of both. DOI: https://doi.org/10.1525/elementa.444.t3

Species (unit)	Stagnation (C1)	Blocking (C2)	Transport-North (C3)	Transport-South (C4)	Non-episodes
		
	May 18–23	June 2, 5, 7, 9, and 10	May 17, 29, and 30	May 25	

O_3_ (ppbv)	38	39	44	59	39
PM_2.5_ (μg m^−3^)	30	29	39	53	27
NO_x_ (ppbv)	48	33	36	16	25
TVOCs (ppbC)	246	162	232	108	155
NO_x_ control^1^	+12%	+9%	+10%	N/A	
VOCs control^2^	−35%	−13%	−26%	N/A	
NO_x_ and VOCs control^3^	−25%	−4%	−17%	N/A	

1 NO_x_,

2 VOCs, and

3 NO_x_ and VOC concentrations were reduced to that of the non-episode.

## Data Availability

Surface data during the KORUS-AQ in South Korea were obtained from the NASA data archive at https://www-air.larc.nasa.gov/cgi-bin/ArcView/korusaq. The cloud coverage and UV data were obtained from the Korea Meteorological Administration website (http://www.data.kma.go.kr).
